# Effect of nattokinase on the pathological conditions in streptozotocin induced diabetic rats

**DOI:** 10.1016/j.heliyon.2024.e28835

**Published:** 2024-03-27

**Authors:** Moe Yamaguchi, Ryo Fukuyama, Mitsugu Fujita

**Affiliations:** Laboratory of Pharmacology, Graduate School of Pharmaceutical Science, Hiroshima International University, 5-1-1 Hirokoshingai, Kure, Hiroshima, 737-0112, Japan

**Keywords:** Nattokinase, Advanced glycation end products, C-Reactive protein, Streptozotocin induced diabetic rat

## Abstract

Nattokinase (NK), also known as subtilisin NAT (EC 3.4.21.62), is a serine protease produced by *Bacillus subtilis natto* that has anti-inflammatory and fibrinolytic properties. To study whether NK prevents the progression of pathological changes in diabetes as an inflammatory disease, we examined the effect of NK on pathological conditions in streptozotocin (STZ)-induced diabetic rats using the following parameters: fasting blood glucose (glucose), total plasma protein (TP), creatinine, histopathology of renal corpuscles and tubules, advanced glycation end products (AGEs), and C-reactive protein (CRP). STZ-administered rats were maintained on a basic diet (CE-2) as control, low-NK diet (containing 0.2 mg NK/g diet), and high-NK diet (0.6 mg NK/g diet) for 14 days. High-dose NK significantly inhibited both glycogen deposition in the renal tubules and increase in the circulating AGE levels without downregulating glucose levels. Compared with the control group, the group treated with the high-NK diet presented a significant inhibition of the increase in the circulating CRP level on day 7 after the beginning of the treatment. However, the CRP level in the NK-H group reached the same level as that in the control group on Day 14. AGEs are known to induce CRP expression in hepatocytes, but the increase in CRP levels in our animal model was independent on the circulating AGE levels. In contrast, low-dose NK did not suppress changes in these parameters. Our present study suggests that NK suppresses glycogen deposition in renal tubules in a dose-dependent manner by the downregulation of AGE formation under hyperglycaemia in the rats with STZ-induced short-term diabetes. However, it is unclear whether this downregulation is caused by intact NK or peptides derived from NK during its digestion in the digestive tract.

## Highlights

• Nattokinase (NK) is a potential preventive supplement for thrombosis and hypertension.
• NK reduces the glycogen deposition in the renal tubules in diabetic rat models.
• NK decreased advance glycation end products (AGEs) and C-reactive protein (CRP) levels in diabetic rat blood.
• This is the first study to demonstrate inhibition of AGE increase by NK in a diabetic model.

## 1 Introduction

Nattokinase (NK) was fined as a fibrinolytic enzyme in 25% v/v ethanol extract of natto, a popular soybean fermented food in Japan [1]. Fujita et al. isolated NK from natto and identified that NK is a new subtilisin-like serine protease with 275 amino acid residues (molecular weight, 27,728) [2]. The cleavage of cross-linked fibrin by NK is six times more efficient than that by plasmin according to the steady-state kinetic parameters for the cleavage of cross-linked fibrin or fibrinogen [3]. Based on these findings, NK has been characterised as a fibrinolytic and anticoagulant agent. NK that injected intravenously dissolved a thrombus more vigorously than plasmin in a chemically induced thrombosis rat model [4] and cleaved the thrombus in a carrageenan-induced thrombosis model [5]. NK increases tissue-type plasminogen activator (tPA) levels in the blood circulatory system by degrading plasminogen activator inhibitor-1 (PAI-1) and releasing tPA from endothelial cells [6,7]. NK is absorbed by the small intestine and cleaves fibrinogen [8], factor VII, and factor VIII [9]. Red blood cell aggregation, blood viscosity, and platelet aggregation are decreased in vitro by NK [10,11].

Recently, Wu et al. evidenced that the anti-thrombotic mechanism of NK is derived from the disruption of the vicious loop among inflammation, oxidative stress, and coagulation [12]. In their study, NK inhibited glomerular fibrin deposition, increased serum PAI-1 levels, and did not affect serum tPA levels during LPS-induced glomerular thrombosis in mice. NK also elicited anti-inflammatory activity by repressing the release of pro-inflammatory mediators from activated macrophages [12].

In recent years, the number of patients with type 2 diabetes mellitus (T2DM) has been increasing posing a serious problem in developed countries, which gives rise to fatal vascular complications [13,14]. Vascular complications, such as retinopathy, nephropathy and atherosclerosis, are caused by endothelial dysfunction during inflammation in T2DM [15].

Therefore, we hypothesised that NK could prevent the progression of pathological conditions by suppressing hyperglycaemia-induced inflammation in T2DM. To test this hypothesis, we investigated the effects of NK on the pathological conditions in rats with streptozotocin (STZ)-induced diabetes.

## 2 Materials and Methods

### 2.1 Materials

Streptozotocin (STZ), diethyl ether, sodium citrate buffer solution (pH 4.5), Cholesterol E-test Wako, and LabAssay™ Triglyceride were purchased from FUJIFILM Wako Pure Chemical (Tokyo, Japan）. Heparin (10,000 units/10 mL) was purchased from Mochida Pharmaceutical (Tokyo, Japan). Pierce™ BCA protein assay kit was purchased from Thermo Fisher Scientific (MA, USA). The Rat C-Reactive Protein ELISA kit was purchased from Bioscience (CA, USA). The creatinine calibrator, DZ072B-CAL, was purchased from Diazyme (CA, USA). OxiSelect™ Advanced Glycation End Product (AGE) Competitive ELISA Kit was obtained from Cell Biolabs (Danvers, MA, USA). SureBeads™ Protein G Magnetic Beads were purchased from Bio-Rad (Hercules, CA, USA).

### 2.2 Nattokinase

NSK-SD was provided by the Japan Bio Science Laboratory (Osaka, Japan) for free, and its properties are as follows: NSK-SD is a spray-dried powder of a compound of the cultural extract with dextrin as the stabiliser. The culture extract was prepared by fermentation with *Bacillus subtilis* natto and then filtered to remove low-molecular-weight impurities. NSK-SD contains NK, a major protein which dominates over 90% of proteins detected by RP-HPLC. The NK content in NSK-SD was 3.4% w/w.

### 2.3 Diets

A regular rodent laboratory diet, CE-2, was purchased from CLEA Japan (Tokyo, Japan) and used as the basic diet. Additionally, CLEA Japan was entrusted with the preparations of the both low- and high-NK diet containing 0.2 and 0.6 mg NK/g, respectively. The diets were prepared to replace 0.6% and 1.8% of CE-2 with NSK-SD, respectively.

### 2.4 Animals

All experimental animal procedures were approved by the Experimental Animal Committee of Hiroshima International University, Hiroshima, Japan (AE14-034). Animal care and use was performed in accordance with the Guide for the Care and Use of Laboratory Animals (National Research Council). Six-week-old male Sprague-Dawley (SD) rats were purchased from Japan SLC (Shizuoka, Japan). The animals were housed three in each cage with the temperature and humidity of the laboratory set at 23 ± 1 °C and 55 ± 5% respectively. The laboratory was maintained in dark from 8:00 p.m. to 8:00 a.m. For 10 days, the animals were given free access to water and CE-2 and allowed to acclimatise to the new environment.

### 2.5 Experimental design

After acclimatisation, the animals were randomly divided into three groups (n = 6 per group) to obtain the average initial fasting blood glucose level and body weight. To induce diabetes, a single intraperitoneal injection of 55 mg/kg/3 mL STZ dissolved in sodium citrate buffer (pH4.5) was administered to each rat on Day 0. The rats in the low-NK diet group (NK-L) and high-NK diet group (NK–H) were fed low-NK diet (0.2 mg/g CE-2) and high-NK diet (0.6 mg/g CE-2) from Day 0 to Day 14, and the rats in a control group (Control) were fed CE-2. Body weight and diet consumption were measured three times per week in each cage that housed three rats each. Fasting blood glucose levels in blood samples (30 mL) collected from the tail vein of each rat, fasted for 13 h, were determined on Day 0 (immediately prior to STZ injection), Day 7, and Day 14 using a glucocard™ MyDIA GT-1670 blood glucose tester (ARKRAY, Japan). After measuring fasting blood glucose levels, blood samples (500 mL) were collected with heparin from the tail vein of each rat anaesthetised with ether to measure total plasma protein (TP), creatinine, C-reactive protein (CRP), and advanced glycation end products (AGEs) level on Days 0, 7, or 14. Blood samples were immediately centrifuged at 2000×*g* for 10 min at 4 °C to obtain the plasma samples. The obtained plasma samples were stored at −80 °C until measurement. At the end of the experimental period (Day 14), the animals were euthanised under anaesthesia by cutting the abdominal aorta, and their left kidneys were removed for histopathological evaluation. In addition to the above protocol, normal kidney and plasma samples to measure AGE levels were obtained from rats (n = 6) in a sham group (Sham) fed CE-2 for 14 days without STZ injection.

### 2.6 Measurement of blood biochemistry

#### 2.6.1 Total plasma protein

TP level in each plasma sample was measured by the bicinchoninic acid (BCA) protein assay with Pierce™ BCA protein assay kits as follows: 25 μL of a diluted plasma sample (120-fold in saline) was mixed with 200 μL of the BCA reagent mixed in 96-well plate and then was incubated for 30 min at 37 °C. Plasma samples were quantified using a microplate reader at 570 nm and a standard curve of bovine serum albumin (kit components).

#### 2.6.2 Creatinine

The creatinine level in each plasma sample was measured using a creatinine calibrator (DZ072B-CAL). Quantification of creatinine in the samples was performed using a microplate reader at 550 nm and with a standard curve of creatinine (kit component).

#### 2.6.3 Advanced glycation end products

AGE levels in each plasma sample were determined using a competitive enzyme-linked immunosorbent assay with the OxiSelect™ AGE Competitive ELISA Kit. In this assay, we used plasma samples diluted 6-fold with saline after the removal of immunoglobulin from each plasma sample with SureBeads™ Protein G Magnetic Beads. AGE levels in plasma samples were measured using a microplate reader at 450 nm using a standard curve of AGE-bovine serum albumin (kit component).

#### 2.6.4 C-reactive protein

CRP level in each plasma sample was measured using a rat C-Reactive Protein ELISA kit. In this assay, plasma samples that were diluted 35000-fold in a buffer containing the kit were used. CRP levels in the plasma samples were measured using a microplate reader at 450 nm (measurement)/570 nm (reference) using the standard curve of rat CRP (kit component).

### 2.7 Histopathological evaluation of kidney

Histopathological evaluation of the kidney was performed by pathologists (Dr. Yasushi Kodama and Dr. Taiki Nishimoto) from the Laboratory of Clinicopathological Therapeutics, Faculty of Pharmaceutical Sciences, Hiroshima International University. Briefly, the kidneys were washed in saline, fixed in 10% formalin neutral buffer solution (pH7.4), dehydrated by using automatic processor, and embedded in paraffin. Paraffin sections (4 mm) were stained with haematoxylin and eosin (HE) and periodic acid-Schiff (PAS) reagents for light microscopy. Histological changes in the renal corpuscles and tubules of the stained sections were evaluated by two pathologists who were blinded to the experimental conditions. Glycogen deposition in the renal tubules of each experimental group was judged from the vacuolisation of tubular epithelial cells and positive PAS staining and was graded into the following five stages: normal, score 0; slight, score 1; moderate, score 2; substantial, score 3; and severe, score 4. The mean score for each group was calculated.

## 3 statistical analysis

All data are expressed as mean ± S.E.M. Comparisons between the two groups were analysed by Student's *t*-test (for parametric data), Welch's *t*-test (for nonparametric data) or the nonparametric Mann–Whitney *U* test. For multiple comparisons, one-way analysis of variance (1-way ANOVA) was performed, and the statistical significance of differences between the control group and each treatment group was analysed using Dunnett's test (for parametric data) or Steel's test (for nonparametric data). Statistical significance was set at *p* < 0.05. Calculations were performed using a commercially available statistical package (Social Survey Research Information Co. Ltd., Tokyo, Japan).

## 4 Results

### 4.1 Data of weight gain, diet consumption, and blood biochemistry

Table 1 presents the average values of weight gain, diet consumption, blood glucose (glucose), TP, and creatinine in rats in each treatment group (Control, NK-L, and NK-H) for 14 days after the beginning of continuous oral administration.

**Table 1:** Weight gain, diet consumption, and blood biochemistry of rats in the study groups.

	Normal (Day 0)	Control	NK-L	NK-H
**Weight gain (g/day)**	–	2.0 ± 0.4	2.0 ± 0.2	2.0 ± 0.2
**Diets consumption (g/body/day)**	–	31.3 ± 1.0	34.1 ± 1.0 equiv. 6.8 mg NK	32.3 ± 1.2 equiv. 19.4 mg NK
**Glucose (mg/dL)**	67.1 ± 1.3	Day 7 360.8 ± 11.7# Day 14 326.8 ± 17.0#	Day 7 369.4 ± 16.7# Day 14 419.8 ± 23.3#	Day 7 398.7 ± 29.2# Day 14 401 ± 31.0#
**TP (μg/mL)**	4.99 ± 0.07	5.20 ± 0.17	5.50 ± 0.08	4.90 ± 0.12
**Creatinine (μmol/L)**	112.2 ± 17.8	160.7 ± 26.0	165.9 ± 16.5	162.2 ± 18.4

The blood glucose (Glucose) of the rats in each group were measured on days 7 and 14 (the end of the experimental period). Total plasma protein (TP) and creatinine levels of rats in each group were measured on day 14. Samples for the measurement of normal values (normal) were obtained from SD rats on day 0 (immediately before STZ injection). Steel's test was used for statistical analysis #*p* < 0.05 versus normal.

The average weight gain in the NK-treatment groups (NK-L and NK-H) was approximately 2.0 g/day and was similar to that in the control group. The dietary consumption of the NK treatment groups was not significantly different from that of the control group. The consumptions of low-NK diet and high-NK diet were 34.1 ± 1.0 g/body/day (the equivalent of 6.8 mg NK/body/day) and 32.3 ± 1.2 g/body/day (the equivalent of 19.4 mg NK/body/day) respectively. All TP levels in the experimental groups were similar to those in the control group. Creatinine levels in the experimental groups were approximately 1.5 times higher than those in the control group and did not significantly change in comparison to the normal group. All glucose levels in the rats injected with STZ on Day 7 were >200 mg/mL. On Day 14, the glucose levels (mg/dL) of the control, NK-L, and NK-H groups were 326.8 ± 17.0, 419.8 ± 23.3, and 401.0 ± 31.0 respectively, and the values were 5–6 times higher than the normal (as of Day 0). However, the glucose levels in the NK-L and NK-H groups were not significantly different from those of the control group. Therefore, NK did not prevent the elevation of glucose levels in STZ-induced diabetic rats.

### 4.2 Histopathological evaluation of kidney

**Fig. 1:**
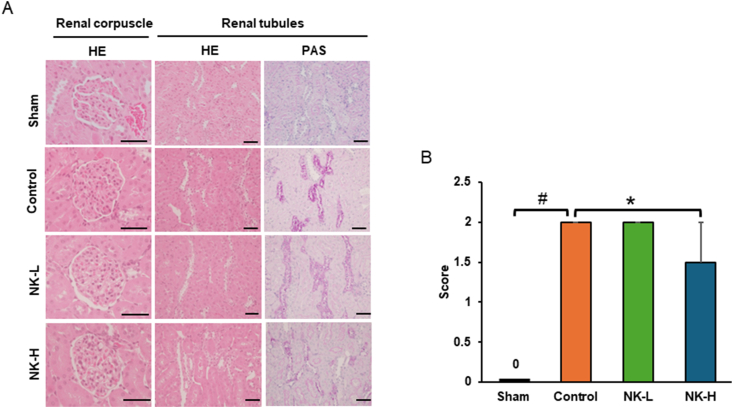
Effect of NK on the histological changes of renal corpuscles or tubules in STZ-induced diabetic rats. A: The rats in the NK-L, NK-H, or control groups were fed low-NK diet (0.2 mg/g CE-2), high-NK diet (0.6 mg/g CE-2), and CE-2, respectively, from Day 0 to Day14 after the injection of STZ. The rats in the sham group (Sham) were fed CE-2 for 14 days without STZ administration. As described in the Materials and Methods, the left kidney in each group was excised, dehydrated, and embedded in paraffin. The paraffin embedded kidneys were thinly sliced (4 μm) and stained with HE and PAS (Scale, 50 μm). B: The glycogen depositions in the renal tubules were semi-quantitatively scored from 0 to 4 as follows; normal: score 0, slight: score 1, moderate: score 2, substantial: score 3, and severe: score 4. The average score of each group was calculated for evaluation. Data are expressed as mean ± S.E.M. Mann–Whitney's *U* test was used for statistical processing. #*p* < 0.05 versus sham, *p < 0.05 versus control.

The renal corpuscles of each experimental group were normally preserved compared to those of the sham group (left lane in Fig. 1A). However, glycogen deposition (PAS-positive area) in the renal tubules of each experimental group was remarkably advanced compared to that in the sham group, and that in the NK-H group was prevented in comparison with that in the control group (Fig. 1A, right). As shown in Fig. 1B, the glycogen deposition score in the renal tubules of the NK-H group was significantly lower than that of the control group. All scores of the rats in the control and NK-L groups were 2 (moderate), whereas those in the NK-H group were half score 1 (slight) and half score 2.

### 4.3 AGE levels

**Fig. 2:**
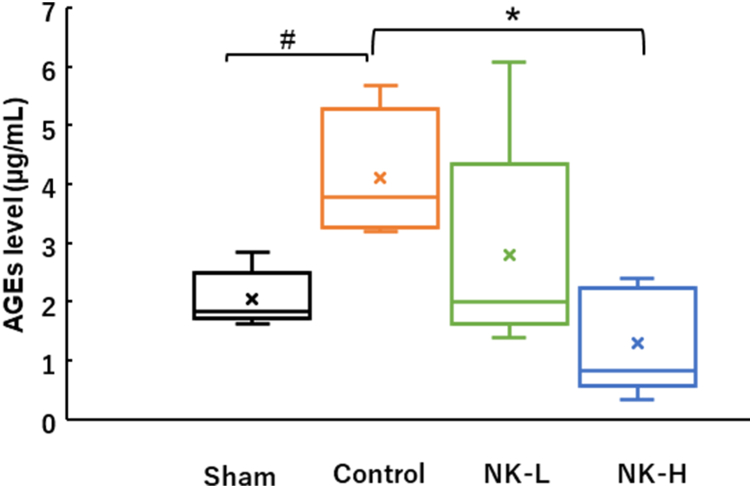
Effect of NK on the elevation of AGE-albumin level in STZ-induced diabetic rats. The rats in the NK-L, NK-H, or control groups were fed low-NK diet (0.2 mg/g CE-2), high-NK diet (0.6 mg/g CE-2), and CE-2, respectively, from Day 0 to Day14 after STZ administration. Rats in the sham group (sham) were fed CE-2 for 14 days without STZ injection. As described in the Materials and Methods section, the circulating AGE-albumin levels in each group on day 14 measured using AGE-competitive ELISA kit. Data are expressed as mean ± S.E.M. #*p* < 0.05 versus sham (Student's *t*-test), *p < 0.05 versus control (Dunnett's test).

As shown in Fig. 2, the AGE level of the control group was 4.21 ± 0.48 μg/dL, and it was significantly increased in comparison with that of the sham group (2.06 ± 0.19 μg/dL). However, AGE levels of the NK-L and the NK-H groups were 2.81 ± 0.75 and 1.30 ± 0.36 μg/dL, respectively, which shows that the latter was significantly lower than that of the control group.

### 4.4 CRP level

**Fig. 3:**
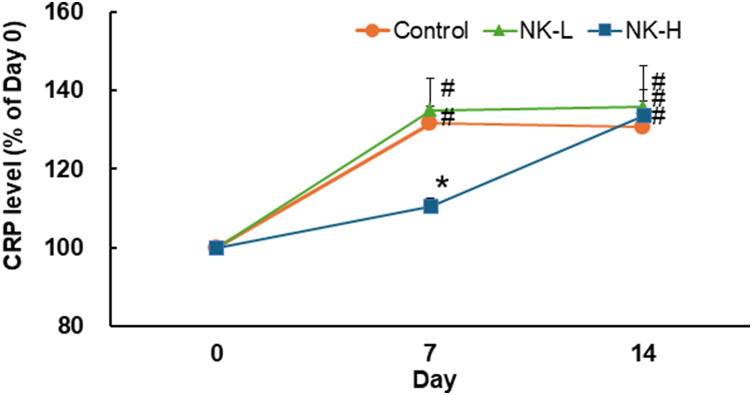
Effect of NK on the elevation of CRP levels in STZ-induced diabetic rats. The circulating CRP levels of the rats in the experimental groups immediately prior to STZ injection (day 0), day 7, and day 14 measured as described in the Materials and Methods. The CRP levels of the experimental groups on days 7 and 14 expressed as percentages relative to the value on day 0 of the paired group. Data are expressed as mean ± S.E.M. #*p* < 0.05 vs day 0 (paired *t*-test), *p < 0.05 vs control on day 7 (Steel's test).

Fig. 3 shows the time-dependent changes in the CRP level in each group. In the control group, the CRP level at day 7 was significantly elevated to approximately 1.3 times as compared with that prior to the STZ injection (day 0) and remained at a high level until day 14. Changes in the CRP levels of the NK-L group were similar to those in the control group. However, the increase in CRP levels in the NK-H group on day 7 was slightly and significantly lower than those in the control group. Thereafter, the CRP levels in the NK-H group reached the same levels as those in the control and NK-L groups on day 14.

## 5 Discussion

STZ is often used to induce diabetes in experimental animals for diabetic animal models as it specifically destroys B cells in the pancreatic islets and decreases insulin secretion. The induction of both types 1 and 2 diabetes in animals is dependent on the STZ dose and the time after STZ injection based on the pathological conditions [16,17]. Animals that lapse 2–4 weeks after a single intraperitoneal injection of 70 mg/kg STZ are commonly used as a type 1 diabetes model. To determine whether NK suppresses the progression of pathological conditions during progression from prediabetes to type 2 diabetes, we assessed the effect of NK on STZ-induced diabetic rats 14 days after STZ injection (STZ-treated short-term diabetic rats). In this study, a low-NK, high-NK, or control diet (CE-2) were orally administered to rats administered 55 mg/kg STZ for 14 days.

Histopathological evaluation was performed around the nephrons of the left kidney that were removed from the rats in each group. The renal corpuscles of each experimental group were preserved for 14 days after STZ injection. As shown in Table 1, the TP and creatinine levels in the experimental groups were not significantly different from those in the normal group. These data indicate that the glomerular function of rats at 14 days after STZ injection was within the normal range.

However, the glycogen deposition in the renal tubules of the control group occurred during the same period after STZ injection. Furthermore, the circulating AGE levels in the control group was approximately 2 times higher than that in the sham group. In the NK-H group, these changes during the 14 days after STZ injection were significantly lower than those in the control group. NK did not downregulate the glucose levels in STZ-induced short-term diabetes. These data indicate that NK suppresses glycogen deposition in the renal tubules depending on the dose and circulating levels of AGEs without downregulating glucose level in STZ-induced short-term diabetes. Diabetic kidney disease (DKD) is triggered by glomerular disorder. Numerous studies using animal models of diabetes have focused on histopathological evaluations of the renal glomeruli. However, Hasegawa et al. revealed that renal tubules were initially damaged by the failure of energy metabolism in the proximal tubules under hyperglycaemia, and then the renal glomeruli were impaired by the discharge of nicotinamide mononucleotide from the damaged tubules [18]. AGEs have been reported to participate in the development and progression of diabetic nephropathy [19,20]. AGEs are filtered from the glomeruli and followed by their uptake into cells by endocytosis via binding to megalin, a membrane receptor on renal tubular epithelial cells [21]. The lysosomal degradation of AGEs reaches its limit in tubular epithelial cells, where AGEs accumulate in tubular epithelial cells to induce tissue damage [22,23]. The formation of AGEs that occurred via formations of Schiff base and Amadori products is prolonged process *in vivo*. However, the formation of AGEs is facilitated by reactive oxygen species (ROS) in Hodge-pathway [24]. In our short-term experiment, the AGEs formation is due to the intracellular ROS generation. Activated macrophages are a source of extracellular AGEs [25], and AGEs from activated macrophages have been targeted for the investigation of therapeutic reagents used to treat diabetes [[26], [27], [28]]. AGEs overproduce intracellular ROS and pro-inflammatory cytokines, such as interleukin-6 (IL-6), interleukin-1b (IL-1b), and tumour necrosis factor a (TNF-a), by binding to the receptor for AGEs (RAGE) and the toll-like receptor 4 (TLR4) on macrophages [29,30] and induce further intracellular AGE formation by a paracrine function. The formation of intracellular AGEs during hyperglycaemia is upregulated by increased intracellular ROS formation [31]. NK reduces the expression of pro-inflammatory mediators (IL-6, TNF-a) and ROS generation in macrophages by suppressing TLR4 and NADPH oxidases 2 (NOX2) activation [12]. These reports support our notion that NK may suppress glycogen deposition in renal tubules via the downregulation of AGEs production by macrophages during hyperglycaemia. However, whether the downregulation of AGE production is caused by intact NK or peptides resulting from NK during digestion in the digestive tract is unclear. Further studies on the effects of intact NK and their peptides on AGE formation by macrophages are warranted.

Diabetes is known to cause complications, such as retinopathy and nephropathy, which are induced by inflammation associated with vascular endothelial dysfunction (angiopathy). Pradhan et al. showed that C-reactive protein (CRP), which is produced by the liver in association with inflammatory events, increases in patients with type 2 diabetes; therefore, inflammation is involved in its development [32,33]. Cho et al. reported that the development of diabetes associated with inflammation can be estimated by CRP levels in the diabetic serum [34]. Circulating CRP is generated in the liver and responds to IL-6, which is enhanced by IL-1b in the acute phase [35,36]. AGEs also directly stimulate CRP production in human hepatoma Hep3B [37]. Hence, we also quantified CRP levels in each group in this experiment. In the NK-H group, the CRP level on Day 7 was lower than that of the control group, and on Day 14 reached the same level as that of the control group during the hyperglycaemic period after 14 days STZ injection. This phenomenon did not coincide with the circulating AGEs level. AGEs are mainly removed from circulation both by filtration from the glomeruli and accumulation in liver. In the NK-H group, the circulating AGEs level on Day 14 may be maintained in normal value in the homeostatic conditions that regulated by not only the removal from circulation but also the downregulation of AGEs production. Consequently, prolongation of AGEs accumulation in liver may delay elevation of the CRP level. In the experiment, association between the AGEs level and the CRP level remains largely undefined. However, Kathryn et al. reported that the serum concentration of AGEs in patients with diabetes was an independent determinant of plasma CRP levels [38]. Therefore, we assume that the increase in CRP levels in our animal model was regulated similar to the above report, not by circulating AGE levels, but by activation of the inflammatory response by AGEs.

In conclusion, NK may be useful for inhibiting AGE formation under hyperglycaemia and suppressing nephropathy progression in diabetes. Our present study suggests that NK suppresses glycogen deposition in the renal tubules in a dose-dependent manner via the downregulation of AGE formation in STZ-treated short-term diabetic rats, which is similar to the progression of pathological conditions in the progression from prediabetes to type 2 diabetes. However, this study is not sufficient to mention the effect of NK on the progression of pathological conditions (e.g. diabetic glomerulosclerosis) in the aggravation of type 2 diabetes, which requires further evaluation using STZ-treated long-term diabetic rats. In addition, whether the downregulation of AGE formation is caused by intact NK or peptides resulting from NK during digestion in the digestive system is unclear. Because the quantitation technique of circulating intact NK or the peptides that administered orally has not yet been developed. Further studies on the effects of intact NK and their peptides on AGE formation by macrophages are warranted.

## Ethics statement

Ethical approval for this study was obtained from Experimental Animal Committee of Hiroshima International University, Hiroshima, Japan (approval No. AE14-034).

## Data availability statement

The data that support the findings of this study are available on request from the corresponding author.

## CRediT authorship contribution statement

**Moe Yamaguchi:** Writing – review & editing, Writing – original draft, Visualization, Methodology, Investigation, Formal analysis, Data curation, Conceptualization. **Ryo Fukuyama:** Methodology, Investigation. **Mitsugu Fujita:** Writing – review & editing, Visualization, Resources, Project administration, Methodology, Investigation, Funding acquisition, Formal analysis, Data curation, Conceptualization.

## Declaration of competing interest

The authors declare the following financial interests/personal relationships which may be considered as potential competing interests: Mitsugu Fujita reports article publishing charges and equipment, drugs, or supplies were provided by Japan Bio Science Laboratory Co., Ltd. Mitsugu Fujita has patent #PCT/JP2024/006164 pending to Japan Bio Science Laboratory Co., Ltd./Josho Gakuen Educational Foundation. Moe Yamaguchi has patent #PCT/JP2024/006164 pending to Japan Bio Science Laboratory Co., Ltd./Josho Gakuen Educational Foundation. If there are other authors, they declare that they have no known competing financial interests or personal relationships that could have appeared to influence the work reported in this paper.
